# Editorial Note

**Published:** 1932

**Authors:** 


### Editorial Note

**The Sheriff of Bristol.**

DR. W. KENNETH WILLS has accepted the office of Sheriff for the coming year, and recalling the admirable manner in which he discharged the duties of President of the Bristol Medico-Chirurgical Society in 1927-8 there is no doubt that the City of Bristol has made a most happy choice. It is many years since a medical man held this important civic office, the late Mr. Richardson Cross having been Sheriff in 1898.

